# Chrysophanol promotes M2 polarization and inhibits M1 polarization through the NF-κB signaling pathway to attenuate sepsis-associated acute kidney injury

**DOI:** 10.3389/fphar.2025.1641068

**Published:** 2025-07-30

**Authors:** Xuan Gou, Wei Zhang, Lele Wang, Caixia Tan, Hong Wei, Xinmin Wang, Le Zhang

**Affiliations:** ^1^College of Medicine, Shihezi University, Shihezi, China; ^2^ Xinjiang Provincial and Ethnic High Incidence Key Laboratory of Ministry of Education, Shihezi, China; ^3^State Key Laboratory of Pathogenesis, Prevention and Treatment of High Incidence Diseasees in Central Asia, Shihezi, China; ^4^ Department of Urology, The First Affiliated Hospital of Shihezi University, Shihezi, China

**Keywords:** chrysophanol, sepsis, acute kidney injury, macrophage polarization, inflammation

## Abstract

**Objective:**

Sepsis-associated acute kidney injury (SA-AKI) is a frequent and severe complication in septic patients. This study combines network pharmacology with *in vitro* and *in vivo* experiments to preliminarily investigate the protective effect of chrysophanol (CHR) on SA-AKI and its mechanism, aiming to find new therapeutic targets and strategies for SA-AKI treatment.

**Methods:**

HK-2 cells were used to investigate CHR’s inhibitory effects on SA-AKI *in vitro* using CCK-8 assay, Hoechst33258 staining, ELISA, Western blot. *In vivo* experiments were performed using a septic mouse model, and the therapeutic effect of CHR on SA-AKI and its effect on macrophage polarization were investigated using Hematoxylin and Eosin staining, ELISA, Western blot, and quantitative real-time PCR. Predicting the possible differentially expressed genes and pathways of CHR protecting SA-AKI through network pharmacology. Finally, these pathways were further validated in *in vitro* experiments by ELISA, Western blot and indirect immunofluorescence staining.

**Results:**

CHR can inhibit LPS-induced injury and apoptosis in HK-2 cells, suppress the expression of inflammatory cytokines TNF-α and IL-6, and enhance its anti-apoptotic and anti-inflammatory effects on HK-2 cells through modulation of macrophages; in *in vivo* experiments, we obtained the same results that CHR effectively counteracted SA-AKI and played a protective role against mice exerting a protective effect. In addition, based on predictions from network pharmacology and validation from cellular experiments, CHR may exert these effects by inhibiting the NF-κB signalling pathway.

**Conclusion:**

CHR may protect SA-AKI by inhibiting the NF-κB signalling pathway, promoting M2 macrophage polarisation and inhibiting M1 macrophage polarisation.

## Graphical Abstract

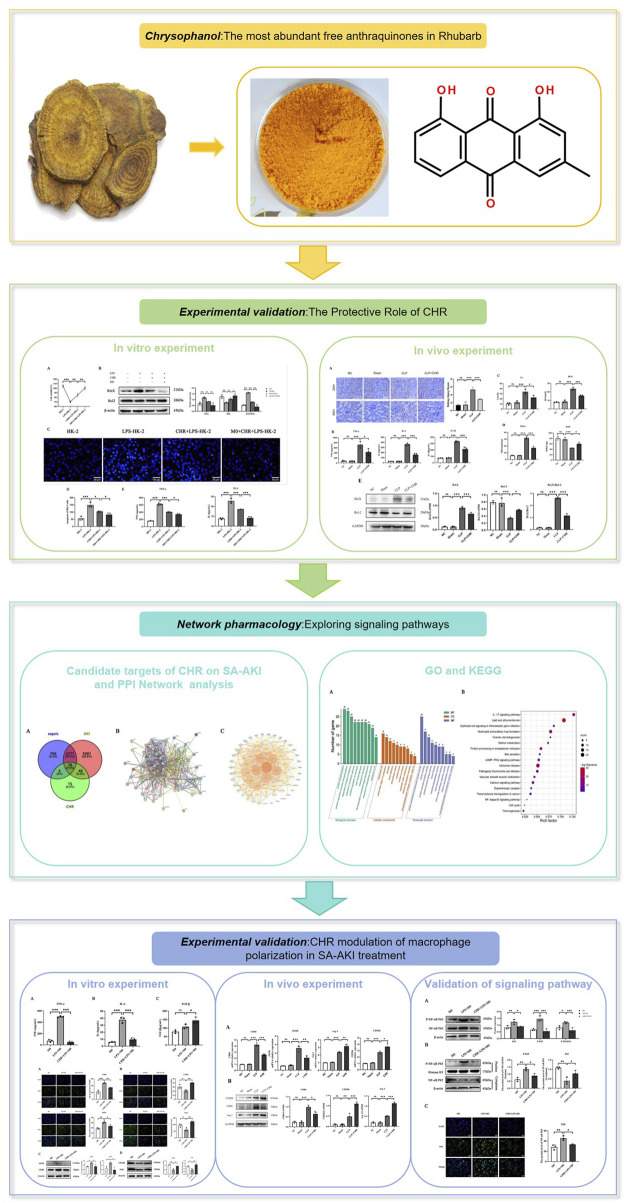

## 1 Introduction

Sepsis is a life-threatening organ dysfunction caused by a dysregulated host response to infection (Font et al., 2020). Acute kidney injury (AKI) develops in approximately 60% of sepsis patients (Wei et al., 2023). This sepsis-associated AKI (SA-AKI) is one of the most common and serious complications of sepsis (Xie et al., 2020), associated with high mortality rates and poor prognosis (He et al., 2022).

The pathological mechanisms underlying SA-AKI remain highly complex and incompletely elucidated. The prevailing view suggests that a cascade of immune-inflammatory responses constitutes the core mechanism driving SA-AKI development (Chang et al., 2022). Within the renal microenvironment, macrophages—as the immune system’s first line of defense—predominantly polarize toward the pro-inflammatory M1 phenotype during early sepsis (Chen et al., 2021). These M1 macrophages upregulate pro-inflammatory mediators (e.g., iNOS, TNF-α, IL-6) (Gui et al., 2020) and activate pathways such as toll-like receptors (TLRs) and nuclear factor-κB (NF-κB), triggering inflammatory cascades that induce renal tissue injury (Zhang and Ning, 2021). Following initial inflammation and injury, the repair phase initiates, during which macrophages shift from the M1 to the anti-inflammatory M2 phenotype. M2 macrophages release anti-inflammatory cytokines (e.g., IL-10), resolving inflammation and promoting tissue repair (Lee et al., 2020). The dynamic balance between M1 and M2 macrophage polarization critically determines inflammatory outcomes and ultimately influences SA-AKI prognosis (Zhang et al., 2024).

SA-AKI poses a significant threat to patient safety. Currently, continuous renal replacement therapy (CRRT) and kidney transplantation represent the only effective treatments; however, clinically proven pharmacological interventions for preventing or treating SA-AKI remain lacking (Romagnoli et al., 2018). Consequently, elucidating the pathogenesis of SA-AKI and developing novel, efficacious therapeutic agents targeting reduced SA-AKI mortality are urgently needed.

*Rhubarb*, derived from the dried rhizomes of *Rheum palmatum* L., *R. tanguticum* Maxim. ex Balf., and *R. officinale* Baill., contains anthraquinones as its primary bioactive constituents (Xu et al., 2024). Clinically, it demonstrates significant efficacy against inflammatory diseases including sepsis (Fang et al., 2007) and chronic kidney injury (CKD) (Tan et al., 2016), promoting restoration of organ structure/function and improving patient survival rates (Xiang et al., 2020). Chrysophanol (CHR), the most abundant free anthraquinone in *Rhubarb*, exhibits lower hepatorenal toxicity (Su et al., 2020), higher bioavailability (Li et al., 2021), and multifaceted therapeutic potential—notably anti-inflammatory, anticancer, and cardioprotective effects (Prateeksha et al., 2019).

Studies indicate CHR attenuates renal inflammation in rat IgA nephropathy models (Gong et al., 2019) and downregulates inflammatory mediators (TNF-α, IL-1β, iNOS, NF-κB) in LPS-stimulated RAW264.7 macrophages (Wen et al., 2020). It similarly ameliorates DSS-induced colitis and LPS-triggered inflammation in murine peritoneal macrophages by suppressing NF-κB activation, IκB-α degradation, and caspase-1 activation (Kim et al., 2010). These findings suggest CHR may mitigate kidney injury through anti-inflammatory mechanisms. Nevertheless, *in vivo* macrophage polarization is multifactorially regulated, and CHR’s specific role in this process—particularly its mechanism of protecting against SA-AKI via macrophage phenotype modulation—remains unexplored.

Current research on Traditional Chinese Medicine (TCM) drugs faces limitations. Thus, this study integrates network pharmacology with *in vitro and in vivo experiments* to investigate CHR’s protective effects against SA-AKI via modulation of macrophage polarization and elucidate its underlying mechanisms.

## 2 Materials and methods

### 2.1 Cells and drugs

HK-2 cells (human renal proximal tubular epithelial cell line) were purchased from Wuhan Procell Life Science and Technology Co., Ltd. and cultured in low-glucose DMEM medium supplemented with 10% fetal bovine serum (FBS) and 1% penicillin-streptomycin. THP-1 cells (human monocytic leukemia cell line) were obtained from Shanghai Institutes for Biological Sciences, Chinese Academy of Sciences and maintained in RPMI-1640 medium containing 10% FBS and 1% penicillin-streptomycin. Both cell lines were incubated at 37°C in a humidified atmosphere with 5% CO_2_. Chrysophanol (CHR) was purchased from Shanghai yuanye Bio-Technology Co., Ltd.

### 2.2 Establishment of SA-AKI cell models and Co-Culture models

HK-2 cells at logarithmic growth phase were trypsinized, resuspended, counted, and seeded in six-well plates. After 24 h of adherence, cells were exposed to 10 μg/mL LPS for 24 h to establish the SA-AKI model.

For THP-1 macrophage differentiation: THP-1 monocytes at logarithmic phase were centrifuged, resuspended, and seeded into Transwell inserts at experimental densities. Cells were induced with 10 ng/mL PMA for 24 h at 37°C/5% CO_2_. Following induction, PMA-containing medium was aspirated. Adherent cells were gently washed twice with PBS along the well walls to remove non-adherent cells and residual agents, yielding M0-polarized macrophages.

For co-culture modeling, LPS-injured HK-2 cells were placed in the lower chamber, while *the PMA-induced M0 macrophages* were transferred to the upper chamber. This configuration simulates the *in vivo* SA-AKI microenvironment.

### 2.3 Animals

Cecal ligation and puncture (CLP) was performed on 6-8-week-old specific pathogen-free (SPF) male C57BL/6 mice weighing 18–25 g. Mice were randomly assigned to four groups (n = 3 per group). The drug intervention group received intraperitoneal injections of 30 mg/kg CHR (Gu et al., 2022) (purity >98%, Henan Skobes Biotechnology Co., Ltd., China; Animal Quality Certificate No. SCXK (Yu) 2020-0005). *For CHR solution preparation, an appropriate quantity of chrysophanol was first dissolved in dimethyl sulfoxide (DMSO) with 0.1% Tween-80, sonicated for 30 min, then diluted in normal saline to a final concentration of 5 mg/mL, and vortex-mixed for 1 h.* All mice had *ad libitum* access to food and water, with bedding replaced every 48 h. Animal experiments were approved by the Science and Technology Ethics Committee of the First Affiliated Hospital, Shihezi University School of Medicine (Approval No. KJ2023-089-01) and complied with the U.S. National Institutes of Health *Guide for the Care and Use of Laboratory Animals* (NIH Publication No. 85-23, revised 1985).

### 2.4 Cell viability assay

HK-2 cells at logarithmic growth phase were seeded in 96-well plates (density: 1 × 10^3^–1 × 10^4^ cells/well). After 24 h exposure to 10 μg/mL LPS, the medium was discarded. Subsequently, PMA-induced THP-1 cells were treated with CHR (0.1, 1, 5, 7.5, 10, 15, or 20 μM) and co-cultured with LPS-injured HK-2 cells for 24 h. Cell viability was assessed by CCK-8 assay.

### 2.5 Hoechst33258 staining

HK-2 cells were seeded on round coverslips and allowed to adhere. After 24 h exposure to 10 μg/mL LPS, the medium was discarded. PMA-induced THP-1 cells were then co-cultured with LPS-treated HK-2 cells in the presence of 10 μM CHR for 24 h. Following co-culture, cells were fixed with 4% paraformaldehyde (1 mL/well, room temperature, 30 min), permeabilized with 0.1% Triton X-100 (5 min), and stained with Hoechst 33,258 (500 μL/well, 5 min). After two gentle PBS washes, coverslips were mounted with antifade mounting medium and imaged under a fluorescence microscope.

### 2.6 ELISA

HK-2 cells at logarithmic phase were seeded in six-well plates and treated with 10 μg/mL LPS for 24 h. After discarding the medium, PMA-induced THP-1 cells were exposed to 10 μM CHR and co-cultured with LPS-injured HK-2 cells for 24 h. Cell culture supernatants were collected, and cytokine levels (TNF-α, IL-6, TGF-β) were quantified using commercial ELISA kits.

For serum analysis, samples from CLP-modeled mice were centrifuged at 3,000 rpm for 15 min. Serum aliquots were assayed immediately after thawing following kit manufacturer protocols. All kits were equilibrated to room temperature for 30 min prior to use.

### 2.7 Western blot

HK-2 cells in the logarithmic growth phase were seeded in six-well plates and exposed to 10 μg/mL LPS for 24 h. After discarding the medium, LPS-treated HK-2 cells were co-cultured for 24 h with PMA-induced THP-1 cells supplemented with 10 μM CHR. Total cellular proteins were then extracted. Nuclear and cytosolic proteins were isolated using a Nuclear and Cytoplasmic Protein Extraction Kit. Protein concentrations were quantified by UV spectrophotometry. Subsequently, proteins were separated by SDS-PAGE and electrotransferred onto polyvinylidene fluoride (PVDF) membranes. Following blocking, membranes were incubated overnight at 4°C with primary antibodies against BAX, Bcl-2, CD86, iNOS, CD206, Arg1, NF-κB p65, phospho-NF-κB p65 (p-p65), and β-actin, followed by 1-h incubation with appropriate HRP-conjugated secondary antibodies (rabbit or mouse IgG).

### 2.8 Animal treatment with CHR

The animal study protocol was approved by the Animal Ethics Committee of Shihezi University School of Medicine. Mice were randomly divided into four groups (n = 3 per group): NC group, Sham group, CLP group, and CLP + CHR group. Following a 12-h fast, mice underwent surgical procedures. Mice in the CLP group were subjected to cecal ligation and puncture (CLP). Mice in the Sham group underwent laparotomy with cecal exteriorization but without ligation or puncture. Immediately after surgery, all mice received a 0.5 mL subcutaneous injection of warm saline on the dorsal surface to prevent shock. Mice in the CLP + CHR group additionally received intraperitoneal injections of CHR (30 mg/kg). Mice in the Sham and CLP groups received equivalent volumes of saline via intraperitoneal injection post-surgery. At 24 h post-surgery, mice were euthanized and samples were collected for subsequent experiments.

### 2.9 Hematoxylin and eosin staining

Kidney specimens from all animals were fixed in 4% paraformaldehyde, embedded in paraffin, and sectioned at 4–6 μm thickness. Sections were stained with hematoxylin and eosin (H&E) for light microscopic examination. Renal histopathological scoring criteria were established as follows: 0 = normal tissue; 1 = 1–25% tubular injury area; 2 = 26–50% injury area; 3 = 51–75% injury area; 4 = 76–100% injury area. For each group, three random fields from H&E-stained kidney sections were evaluated in a blinded manner to assess renal tubular damage and quantify the affected area.

### 2.10 RNA extraction and reverse transcription-quantitative polymerase Chain reaction (RT-qPCR)

Total RNA was extracted using TRIzol reagent according to the manufacturer’s instructions. Equal amounts of total RNA were treated with gDNA Eraser reagent to eliminate potential genomic DNA and used for cDNA synthesis in a 20 μL reaction system. RT-qPCR amplification analysis was performed using SYBR Green Master Mix for q225 using 2 μL of cDNA.

Primer sequence:

iNOS  forward:5′-TTCAGTATCACAACCTCAGCAAG,
    reverse:5′-TGGACCTGCAAGTTAAAATCCC,
CD86  forward:5′-CTGCTCATCTATACACGGTTACC,
    reverse:5′-GGAAACGTCGTACAGTTCTGTG,
GAPDH  forward:5′-ACAACAGCCTCAAGATCATCAGC,
    reverse:5′-GCCATCACGCCACAGTTTCC.

### 2.11 Immunofluorescence

HK-2 cells were seeded on round coverslips and allowed to adhere. After 24 h exposure to 10 μg/mL LPS, the medium was discarded. PMA-induced THP-1 cells were co-cultured with LPS-injured HK-2 cells in 10 μM CHR-containing medium for 24 h. Following co-culture, coverslips were washed with PBS and fixed with 4% paraformaldehyde (10 min, RT), then permeabilized with 0.2% Triton X-100 (15 min, RT). After blocking with 5% BSA (30 min, RT), cells were incubated with primary antibody overnight at 4°C in a humidified chamber. Coverslips were washed thrice with PBS, incubated with fluorophore-conjugated secondary antibody (2 h, RT, light-protected), and counterstained with DAPI (5 min, RT, dark). Finally, coverslips were mounted with antifade medium and imaged under an inverted fluorescence microscope. Image acquisition and semi-quantitative analysis were performed using ImageJ.

### 2.12 Acquisition of CHR, sepsis and acute kidney injury targets

Potential therapeutic targets of chrysophanol (CHR) were identified by querying TCMSP, SwissTargetPrediction, and Comparative Toxicogenomics Database (CTD) using “chrysophanol” as the key search term. Retrieved protein targets were standardized to official gene symbols via UniProt (Daina et al., 2019). Sepsis and acute kidney injury (AKI)-associated targets were acquired from DisGeNET, GeneCards, and Online Mendelian Inheritance in Man (OMIM) databases.

### 2.13 Target screening for CHR treatment of acute SA-AKI

Common targets of chrysophanol (CHR) and sepsis/acute kidney injury (AKI) were identified using Venny 2.1 by intersecting CHR-associated targets with disease-associated targets. These overlapping targets represent potential therapeutic targets for CHR in SA-AKI treatment.

### 2.14 Protein-protein interaction network construction (PPI)

Common targets of chrysophanol (CHR) and sepsis-associated acute kidney injury (SA-AKI) were analyzed using the STRING database for protein-protein interactions (PPIs). The PPI network data was exported in TSV format and imported into Cytoscape for visualization. Core therapeutic targets were identified through topological analysis, ranked by degree centrality using CytoHubba.

### 2.15 GO and KEGG enrichment analysis

Gene Ontology (GO) and Kyoto Encyclopedia of Genes and Genomes (KEGG) enrichment analyses of common targets were performed using Metascape, encompassing biological processes (BP), molecular functions (MF), cellular components (CC), and signaling pathways to elucidate potential mechanisms underlying CHR’s therapeutic effects against SA-AKI.

### 2.16 Statistical analysis

Statistical analyses were conducted using SPSS version 20.0. Data were presented as mean ± standard deviation. Differences between groups were compared using the *t*-test. Univariate and multi-variate ANOVAs were used for multiple-group comparisons. Statistical significance versus the control group was defined as *p* < 0.05, *p* < 0.01 or *p* < 0.001. Statistical plots were generated using GraphPad 9.3. All experiments were repeated at least three times.

## 3 Results

### 3.1 Protective effect of CHR on SA-AKI

#### 3.1.1 Effect of CHR on HK-2 cell viability in different groups

**FIGURE 1:**
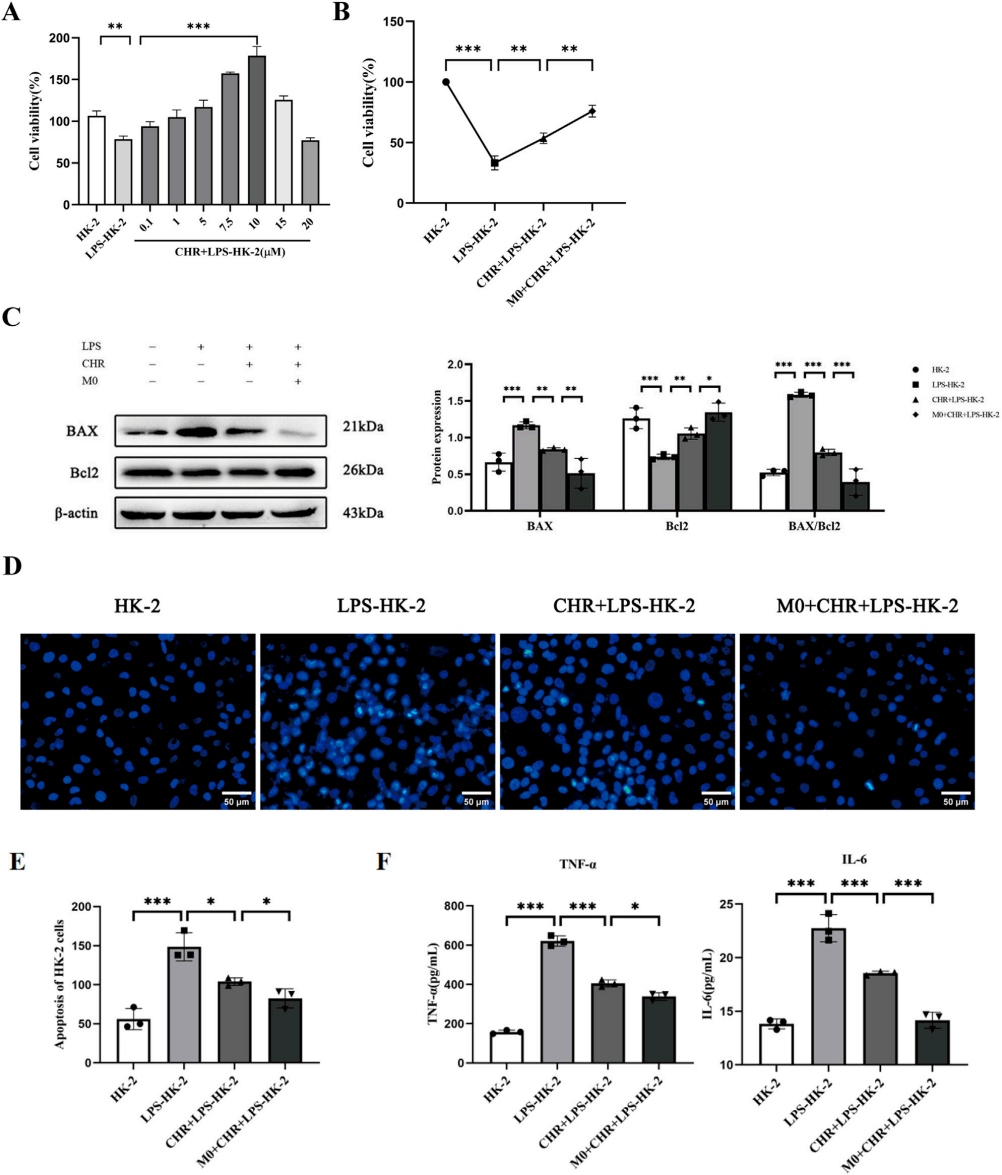
Effect of CHR on SA-AKI in HK-2 cells. **(A,B)** Effect of CHR on the viability of HK-2 cells in each group. **(C)** WB detection of apoptosis-related protein expression. **(D,E)** Hoechst 33,258 staining method to detect morphological changes of apoptosis in each group of HK-2 cells (×400). **(F)** Expression of supernatant inflammatory factors TNF-α and IL-6 in each group of HK-2 cells. Data are presented as mean ± standard deviation (*p < 0.05, **p < 0.01, ***p < 0.001).

The CCK-8 assay showed significantly reduced viability in LPS-treated HK-2 cells versus controls (*p* < 0.01), establishing an *in vitro* SA-AKI model. CHR treatment increased cell viability concentration-dependently, with maximal viability observed at 10 μM (*p* < 0.001). At 15 μM, viability decreased compared to the LPS group. Therefore, 10 μM CHR was selected for subsequent experiments (Figure 1A). Further experiments confirmed LPS-induced viability loss (*p* < 0.001). CHR restored viability after LPS injury (*p* < 0.01), and enhanced protection was observed in co-culture (*p* < 0.01) (Figure 1B), indicating macrophage-mediated protection.

Collectively, these findings demonstrate that CHR exerts protective effects on HK-2 cells through macrophage polarization modulation.

#### 3.1.2 Effect of CHR on apoptosis-related proteins in HK-2 cells

The decreased BAX/Bcl-2 ratio indicates inhibition of apoptosis. This implies that CHR inhibits LPS-induced apoptosis in HK-2 cells and enhances anti-apoptotic effects on HK-2 cells through macrophage modulation (Figure 1C).

#### 3.1.3 Effects of CHR on apoptotic morphology of HK-2 cells

In Figure 1D, control HK-2 cells exhibited uniform diffuse blue fluorescence. The LPS-treated group showed distinct apoptotic features, including dysmorphic cellular morphology, increased debris, and densely stained granular nuclear fluorescence (*p* < 0.001). Compared to the LPS group, the CHR + LPS group demonstrated significantly reduced apoptotic cells (*p* < 0.05), indicating CHR’s inhibition of LPS-induced damage and apoptosis. Furthermore, the M0 co-culture + CHR + LPS group showed reduced apoptosis versus the CHR + LPS group (*p* < 0.05), confirming enhanced anti-apoptotic effects through macrophage modulation (Figure 1E).

#### 3.1.4 Effect of CHR on the expression of inflammatory factors in HK-2 cells

To further investigate CHR’s effects on inflammatory factors, ELISA quantified cytokine expression in cell supernatants (Figure 1F). Results demonstrated that CHR inhibited TNF-α and IL-6 release in LPS-injured HK-2 cells (*p* < 0.001) and enhanced this anti-inflammatory effect through macrophage modulation (*p* < 0.05).

#### 3.1.5 Effect of CHR on SA-AKI in mice

**FIGURE 2:**
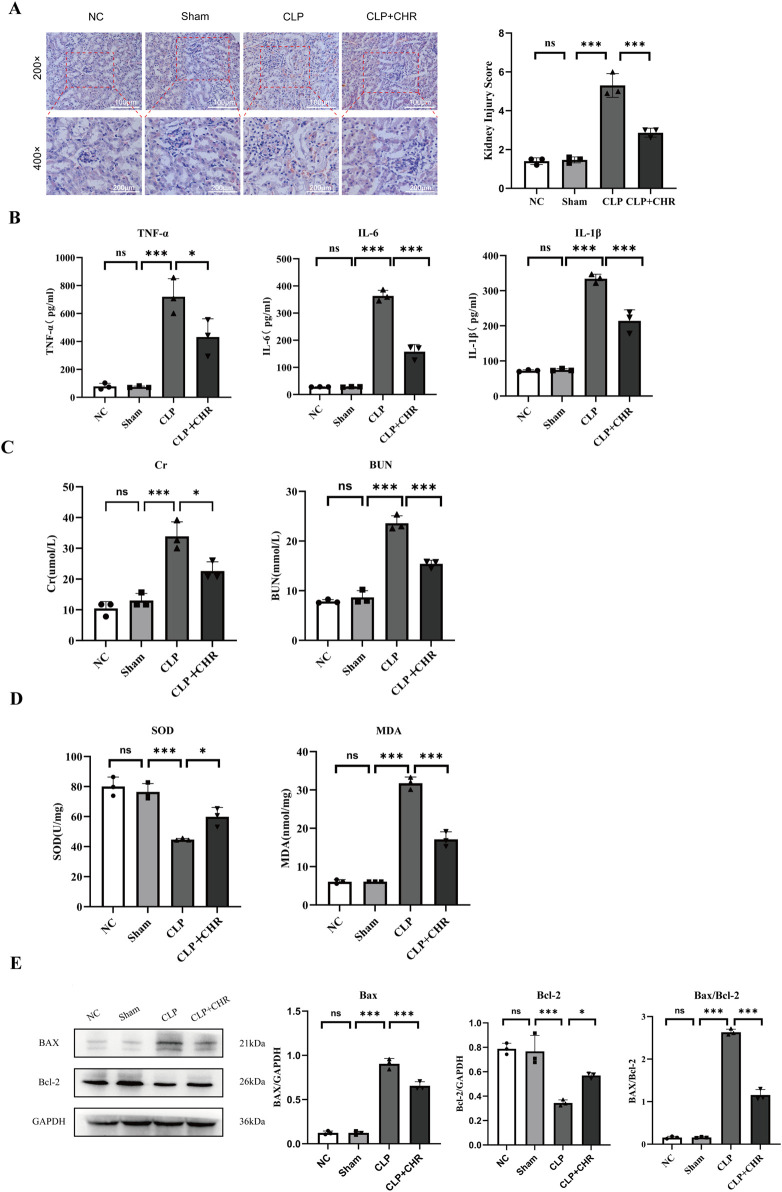
Effect of CHR on SA-AKI in mice. **(A)** HE images and damage scores of mouse kidney tissue. **(B)** Expression of inflammatory factors TNF-α, IL-6 and IL-1β in each group. **(C)** Expression of renal function (Cr, BUN) in each group **(D)** Expression of oxidative stress levels (MDA, SOD) in each group **(E)**Expression of apoptotic proteins (BAX, Bcl-2) in each group Data are presented as mean ± standard deviation (*p < 0.05, ***p < 0.001)

Histological and renal functional alterations aligned with *in vitro* findings. NS and Sham groups showed no significant differences. The CLP group exhibited severe renal injury (*p* < 0.001), characterized by vacuolar degeneration and detachment of renal tubular epithelial cells (Figure 2A). Serum analysis revealed elevated inflammatory factors (Figure 2B): TNF-α, IL-6, and IL-1β (*p* < 0.001), alongside increased creatinine (Cr) and blood urea nitrogen (BUN) (*p* < 0.001) (Figure 2C). CHR treatment ameliorated renal damage versus CLP (*p* < 0.001), reducing TNF-α, IL-6, IL-1β, Cr, and BUN (*p* < 0.05). Oxidative stress analysis (Figure 2D) showed elevated malondialdehyde (MDA) (*p* < 0.001) and reduced superoxide dismutase (SOD) (*p* < 0.001) in CLP group. CHR reversed these trends (MDA: *p* < 0.001; SOD: *p* < 0.05). Western blot analysis (Figure 2E) demonstrated decreased pro-apoptotic BAX (*p* < 0.001) and increased anti-apoptotic Bcl-2 (*p* < 0.05) in CHR-treated mice. The reduced BAX/Bcl-2 ratio confirms CHR’s anti-apoptotic protection against SA-AKI.

### 3.2 Network pharmacology

#### 3.2.1 Acquisition of CHR, sepsis and acute kidney injury targets

After deduplication, 145 unique CHR targets were identified from SwissTargetPrediction, TCMSP, and CTD. Separately, 3,219 sepsis-related and 7,578 AKI-related targets were obtained from DisGeNET, GeneCards, and OMIM following duplicate removal.

#### 3.2.2 Screening of targets of CHR for the treatment of SA-AKI

**FIGURE 3:**
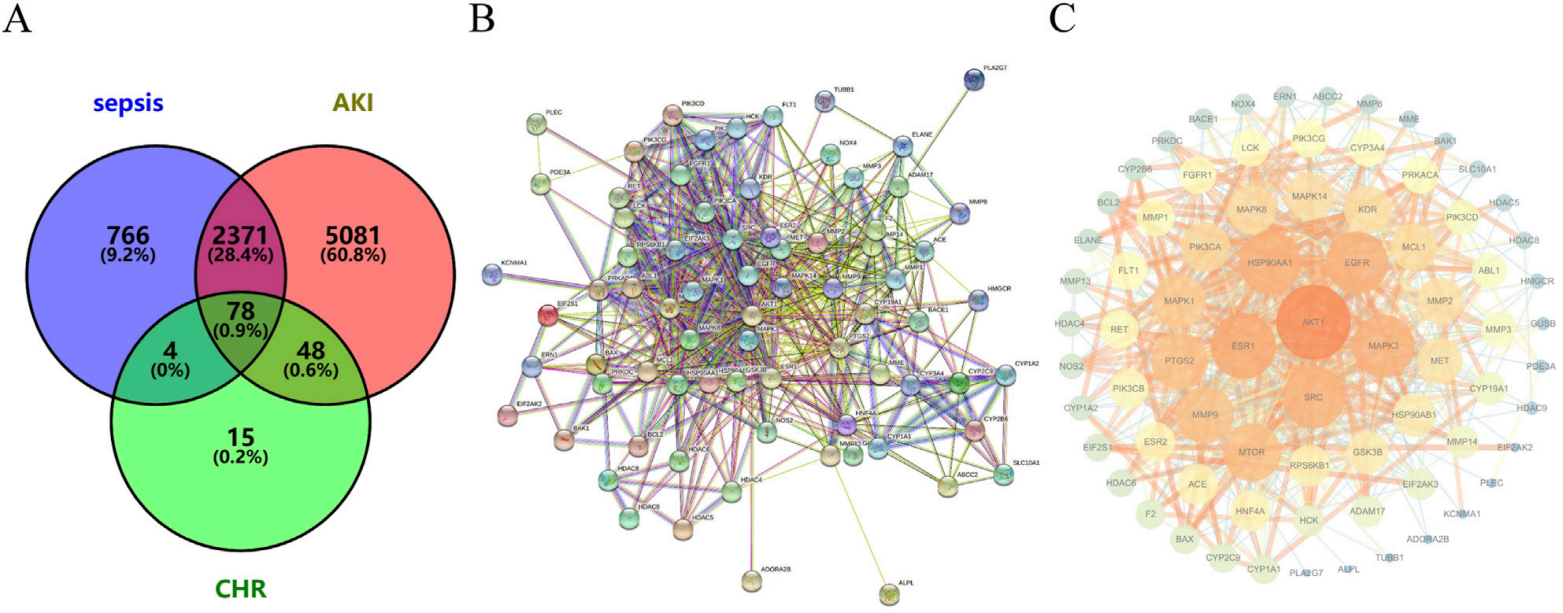
CHR and SA-AKI targets **(A)** Venn diagram of genes intersecting the targets of action of CHR and SA-AKI. **(B)** PPI network map of the core target intersection genes of CHR and SA-AKI. **(C)** Ranking the core targets of CHR for the treatment of SA-AKI according to degree values.

Venny 2.1.0 intersection analysis of CHR targets and sepsis-associated acute kidney injury (SA-AKI) targets identified 78 potential therapeutic targets for CHR in SA-AKI treatment (Figure 3A).

#### 3.2.3 Construct PPI network

The PPI network was constructed using STRING 11.0 and visualized in Cytoscape. Topological analysis revealed key targets ranked by degree centrality (Figures 3B,C), where higher degree values indicate greater node connectivity, stronger protein interactions, and broader functional involvement. Core targets for CHR in SA-AKI treatment included AKT1, ESR1, HSP90AA1, EGFR, and MAPK3.

#### 3.2.4 GO and KEGG enrichment analysis

**FIGURE 4:**
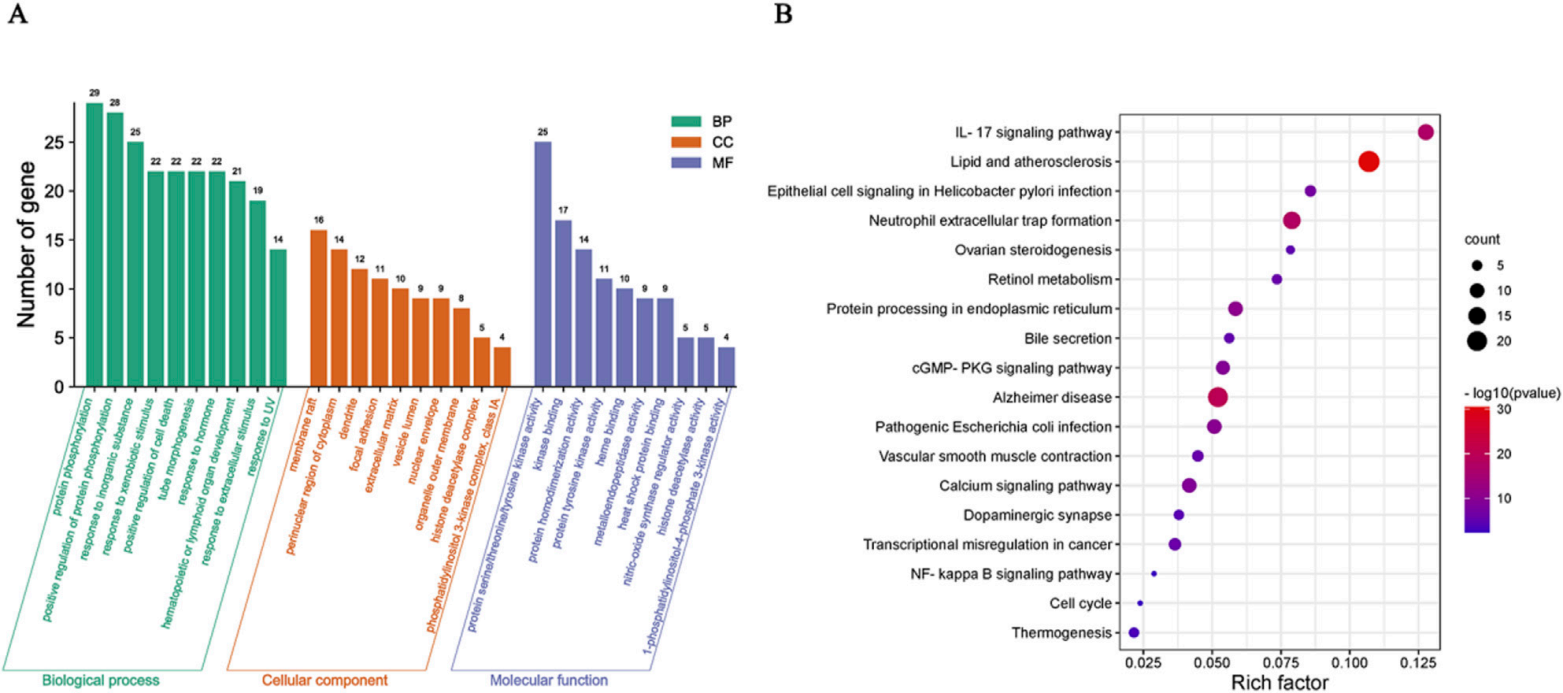
Enrichment analysis of CHR and SA-AKI. **(A)** Results of GO pathway enrichment analysis. **(B)** Results of KEGG pathway enrichment analysis.

The results of GO and KEGG enrichment analysis showed that CHR may treat SA-AKI through multiple pathways and targets (Figure 4). These analyses revealed the involvement of key signaling pathways, including IL-17 signaling pathway, cGMP-PKG signaling pathway, Calcium signaling pathway, and NF-κB signaling pathway. Among them, NF-κB signaling pathway closely associated with both the inflammatory response and macrophages and linked to core targets. Integrating the key target prediction and KEGG pathway enrichment analysis, it was hypothesized that the NF-κB signaling pathway was a key pathway for CHR in the treatment of SA-AKI. Therefore, the NF-κB signaling pathway was selected in the present study for subsequent experimental validation.

### 3.3 CHR modulation of macrophage polarization in SA-AKI treatment

#### 3.3.1 ELISA for the expression of polarization-related factors in macrophages

**FIGURE 5:**
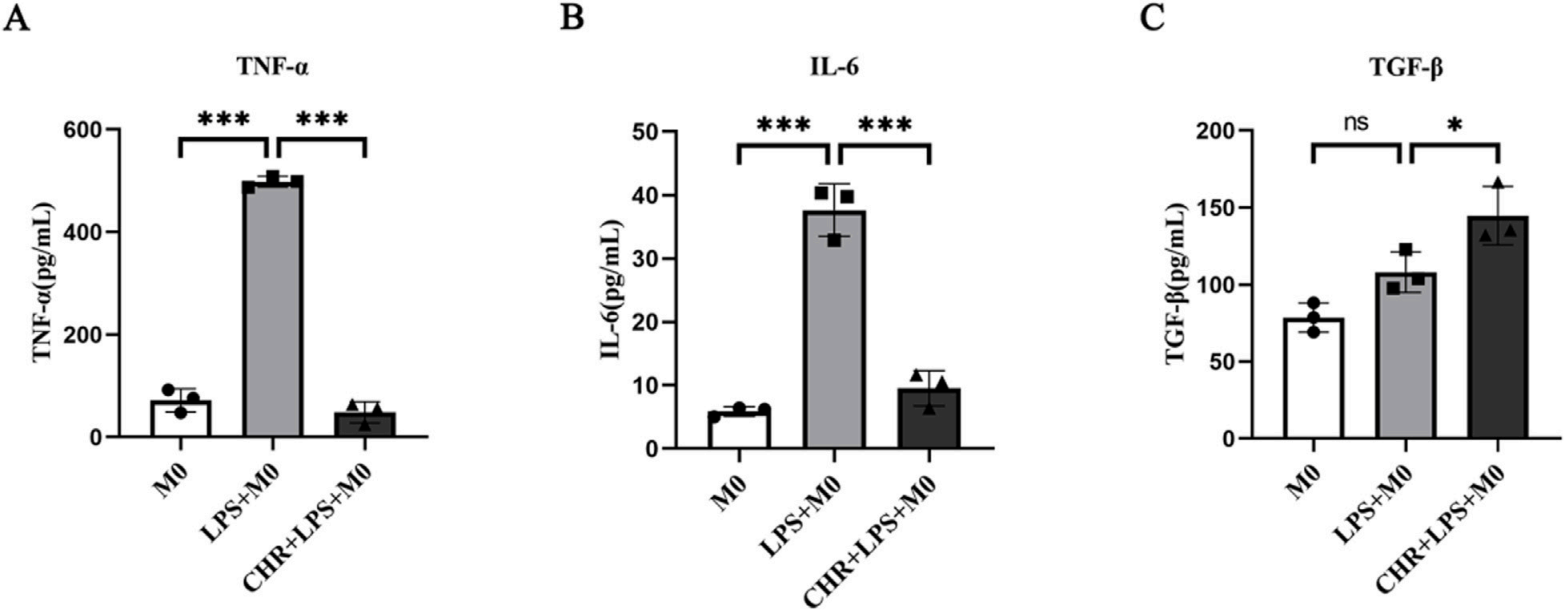
Expression of **(A)** TNF-α, **(B)** IL-6and **(C)** TGF-β, the polarization-related factors in macrophage supernatants. Data are presented as mean ± standard deviation (*p < 0.05, **p < 0.01, ***p < 0.001).

To further investigate the effect of CHR on macrophages, THP-1 cells were treated with 10 μg/mL LPS for 24 h, followed by 10 μM CHR for an additional 24 h. Cell supernatants were then collected, and secretion of polarization-associated factors was measured by ELISA (Figure 5). In the LPS group, levels of M1 macrophage polarization markers TNF-α and IL-6 were significantly elevated (*p* < 0.001). CHR intervention decreased TNF-α and IL-6 expression (*p* < 0.001) while increasing expression of the M2 macrophage polarization marker TGF-β (*p* < 0.05). These findings indicate that CHR inhibits M1 macrophage polarization and promotes polarization toward M2-type macrophages.

#### 3.3.2 IF and WB for the expression of macrophage polarization-related proteins

**FIGURE 6:**
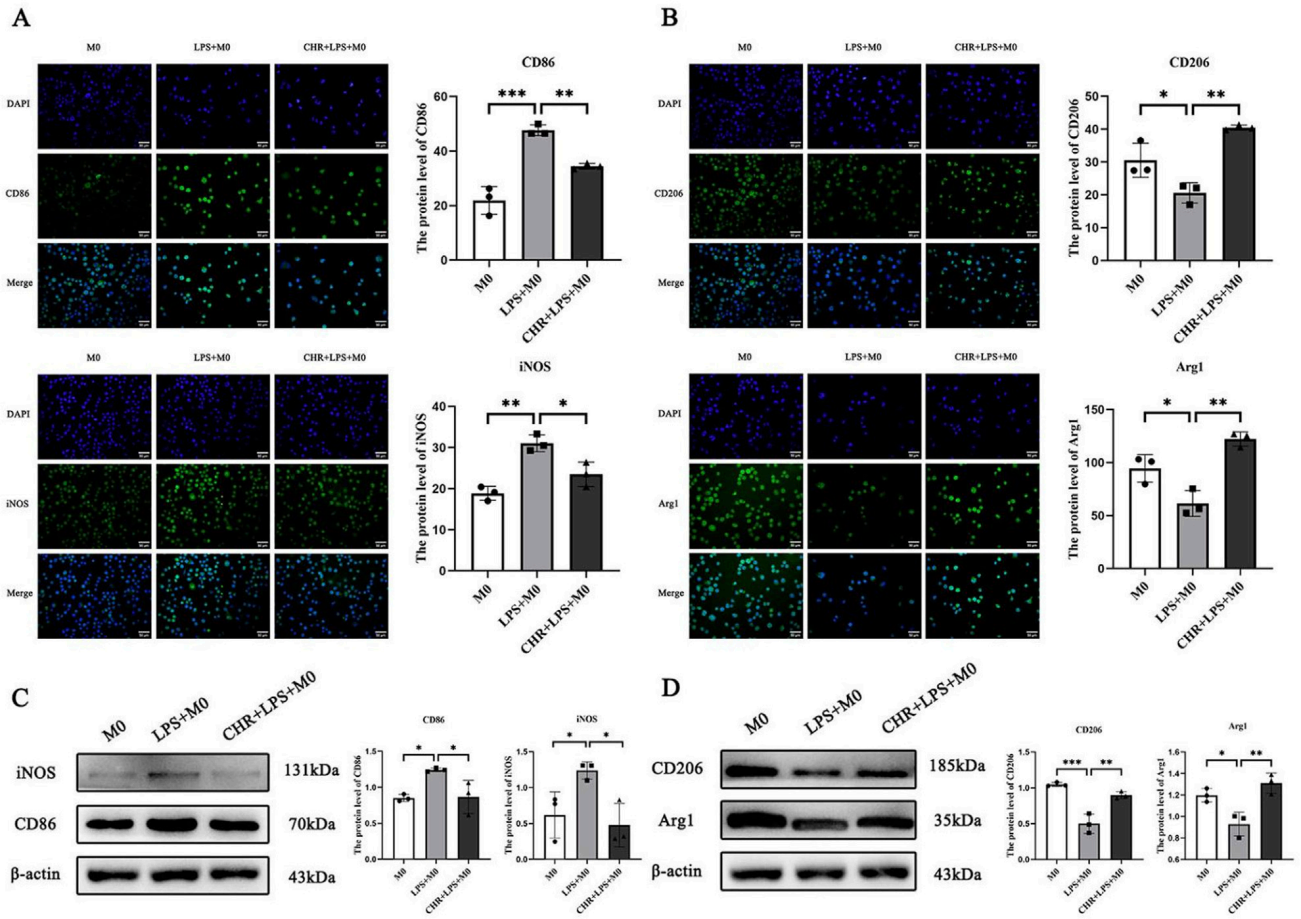
The expression of macrophage polarization-related proteins. IF detection of **(A)** macrophage M1 polarization-related protein CD86, iNOS expression and **(B)** macrophage M2 polarization-related protein CD206, Arg1 expression. WB detection of **(C)** macrophage M1 polarization-related protein CD86, iNOS expression and **(D)** macrophage M2 polarization-related protein CD206, Arg1 expression. Data are presented as mean ± standard deviation (*p < 0.05, **p < 0.01, ***p < 0.001).

In the LPS group, levels of M1 macrophage polarization markers CD86 and iNOS were significantly increased (*p* < 0.01). Their expression decreased in the CHR-treated group compared to the LPS group (*p* < 0.05), indicating that LPS induced M1 macrophage polarization and CHR intervention inhibited this process (Figures 6A,C).

Levels of M2 macrophage polarization markers CD206 and Arg-1 were reduced in the LPS group (*p* < 0.05) but increased in the CHR-treated group (*p* < 0.01). This demonstrates that CHR promoted macrophage polarization toward the M2 phenotype (Figures 6B,D).

#### 3.3.3 qRT-PCR and WB for the expression of macrophage polarization-related proteins in mice *in vivo*

**FIGURE 7:**
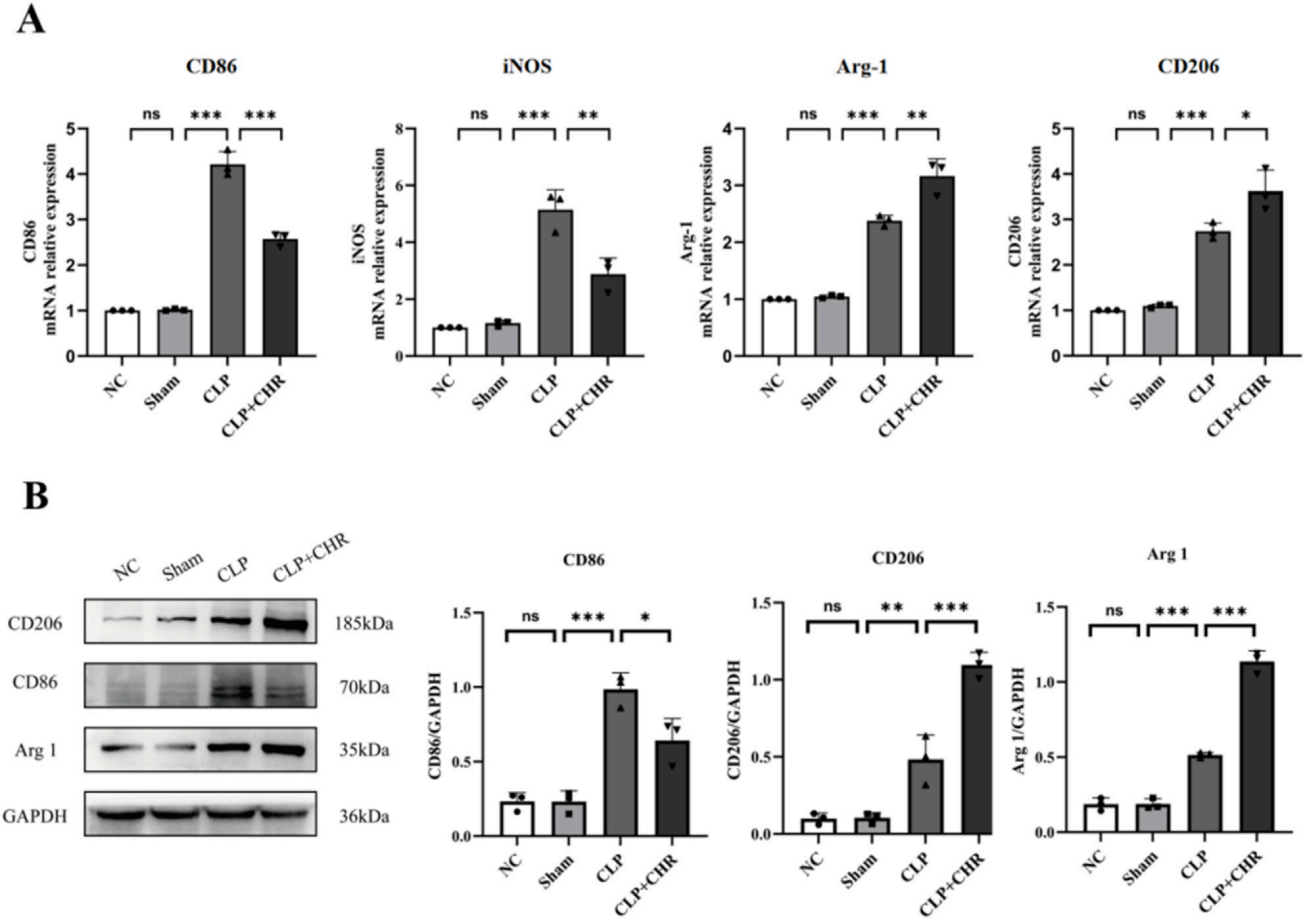
The expression of polarization-related proteins in mouse macrophages. **(A)** qRT-PCR detection of macrophage M1 polarization-associated genes iNOS and CD86 and M2 polarization-associated genes Arg-1 and CD206 expression. **(B)** WB detection of macrophage M1 polarization-related protein CD86 expression and macrophage M2 polarization-related protein CD206, Arg1 expression. Data are presented as mean ± standard deviation (*p < 0.05, **p < 0.01, ***p < 0.001).

We subsequently examined macrophage polarization markers in kidney tissues from each mouse group using qRT-PCR (Figure 7A) and Western blotting (Figure 7B). Compared with the control group, levels of M1-type polarization markers iNOS and CD86 were significantly elevated in CLP group renal tissues (*p* < 0.001). CHR intervention significantly reduced iNOS and CD86 expression (*p* < 0.01) while increasing levels of M2-type polarization markers Arg-1 and CD206 (*p* < 0.05). These findings were consistent with Western blot results. Collectively, the data indicate that macrophages in CLP group kidneys exhibited M1 polarization, whereas CHR inhibited M1 polarization and promoted polarization toward the M2 phenotype.

#### 3.3.4 IF and WB for the expression of NF-κB signaling pathway-related proteins in macrophages

While the above findings suggest that CHR alleviates SA-AKI by regulating macrophage polarization, further experiments are required to elucidate the underlying mechanisms. Network pharmacology analysis suggested that the NF-κB signaling pathway may represent the central mechanism mediating CHR’s therapeutic effects in SA-AKI and is critically involved in polarization regulation. Therefore, we detected expression of NF-κB pathway-related proteins in macrophages by Western blotting.

**FIGURE 8:**
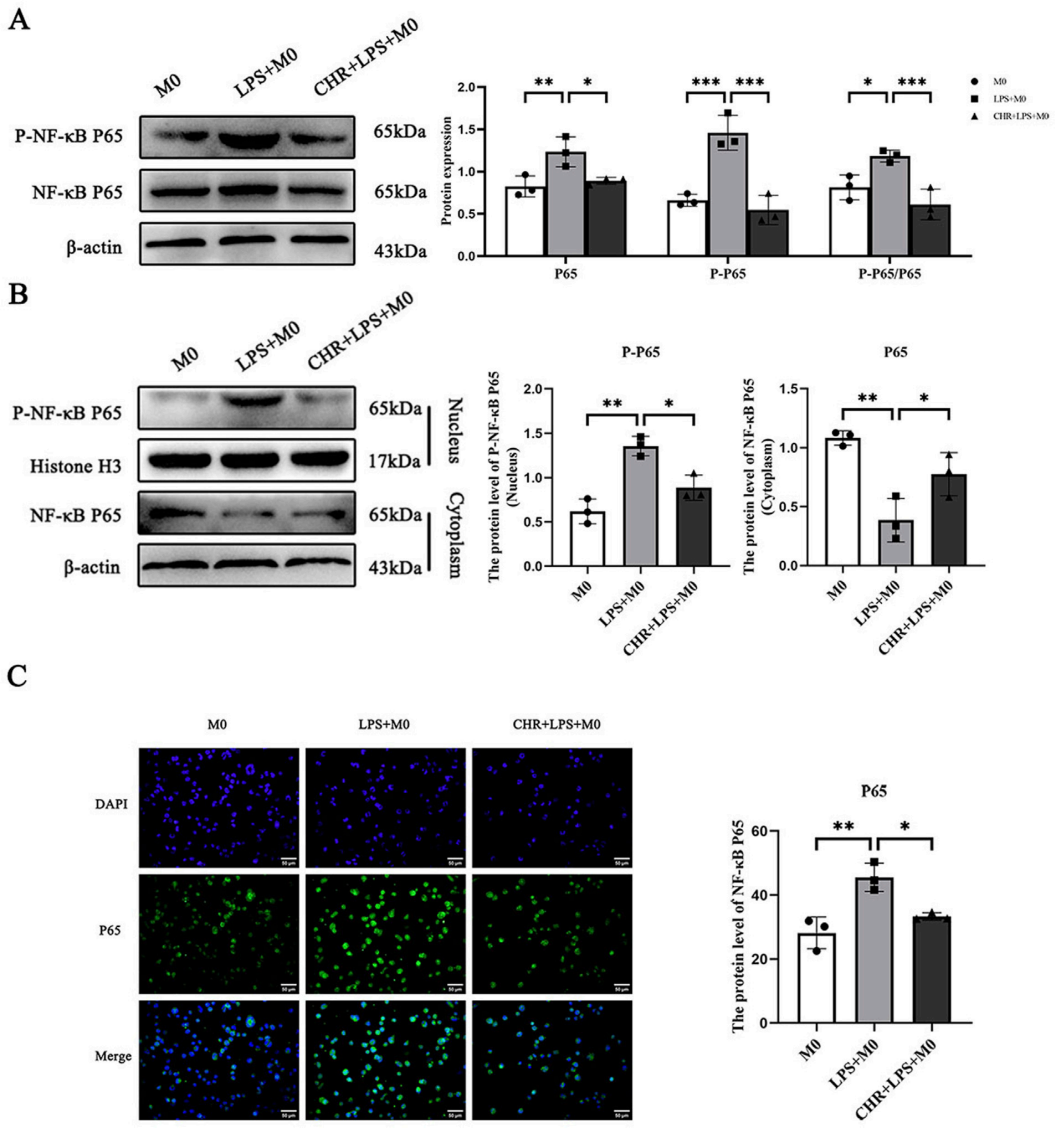
The expression of NF-κB signaling pathway-related proteins. **(A)** WB detection of protein expression of NF-κB P65, P-NF-κB P65 in total protein. **(B)** The protein expression of P-NF-κB P65 in nuclear proteins and NF-κB P65 in plasma proteins. **(C)** IF detection of protein expression levels of nuclear NF-κB P65/total NF-κB P65. Data are presented as mean ± standard deviation (*p < 0.05, **p < 0.01, ***p < 0.001).

Using M0 macrophages as negative controls, we treated cells with LPS or CHR + LPS. Immunoblotting analysis revealed that in whole-cell lysates (Figure 8A), expression of NF-κB p65 and phospho-NF-κB p65 (p-p65) was elevated in the LPS group (*p* < 0.01), with increased p-p65/p65 ratio (*p* < 0.05). Conversely, CHR co-treatment reduced p-p65 expression (*p* < 0.05) and significantly decreased p-p65/p65 ratio (*p* < 0.001).

Nuclear-cytoplasmic fractionation analysis (Figure 8B) demonstrated elevated nuclear p-p65 (*p* < 0.01) and reduced cytoplasmic NF-κB p65 (*p* < 0.01) in LPS-treated cells. CHR intervention decreased nuclear p-p65 (*p* < 0.05) while increasing cytoplasmic p65 (*p* < 0.05). These results indicate that LPS activates the NF-κB pathway by promoting p65 phosphorylation and nuclear translocation, whereas CHR inhibits LPS-induced NF-κB activation.

Immunofluorescence (IF) analysis revealed predominant cytoplasmic NF-κB p65 expression in control macrophages (Figure 8C). LPS treatment significantly increased the nuclear-to-total NF-κB p65 ratio (*p* < 0.01), indicating NF-κB pathway activation and enhanced p65 nuclear translocation. Conversely, CHR co-treatment reduced the nuclear p65 ratio versus LPS (*p* < 0.05), demonstrating inhibition of LPS-induced p65 nuclear translocation.

## 4 Discussion

Sepsis is currently defined by Sepsis-3 criteria as a life-threatening organ dysfunction caused by a dysregulated host response to infection (Shankar et al., 2016). Patients with sepsis-associated acute kidney injury (SA-AKI) represent one of the most severe complications of sepsis. To date, no specific therapies exist for SA-AKI beyond renal replacement therapy or kidney transplantation.

The pathogenesis of sepsis-associated acute kidney injury (SA-AKI) is highly complex. Current evidence identifies an overactive immune response and cytokine storm as direct contributors to SA-AKI (Fernández et al., 2015). Macrophages, key innate immune cells, play a crucial role in the development of SA-AKI (Kadomoto et al., 2021) and are involved throughout the inflammatory response, from its initiation to resolution (Rodríguez-Morales and Franklin, 2023). Stimulated by microenvironmental factors, macrophages polarize into distinct phenotypes, primarily classified as classically activated (M1) or alternatively activated (M2), each fulfilling specific functions (Wang et al., 2021). M1 macrophages, classically activated by lipopolysaccharide (LPS) and interferon-gamma (IFN-γ), secrete pro-inflammatory cytokines (Bashir et al., 2016). In contrast, M2 macrophages promote tissue repair and immune tolerance (Shapouri-Moghaddam et al., 2018). suppressing inflammation through the secretion of interleukin-10 (IL-10), arginase-1 (ARG), and transforming growth factor-beta (TGF-β) (Bashir et al., 2016).

Pharmacokinetic studies indicate that CHR, a naturally occurring anthraquinone, exhibits enhanced cellular absorption, superior bioavailability, and reduced hepatorenal toxicity compared to other free anthraquinones (Xie et al., 2019). These properties underscore its therapeutic promise for diverse pathological conditions.

Furthermore, CHR protects against sepsis-induced acute myocardial injury by attenuating inflammation and cardiomyocyte apoptosis via the miR-27b-3p/PPAR-γ pathway (Zhao et al., 2022), suggesting its potential to safeguard organs from sepsis-induced damage through anti-inflammatory modulation. Consistent with this mechanism, CHR attenuates LPS-induced inflammation in macrophages by suppressing NF-κB activation in a PPAR-γ-dependent manner (Wen et al., 2018), thereby counteracting inflammatory responses. Additionally, CHR protects against progressive kidney injury by regulating apoptosis and endoplasmic reticulum stress (Lin et al., 2021).

In the present study, we demonstrated that CHR significantly attenuated SA-AKI progression in both *in vivo* and *in vitro* models. A cellular SA-AKI model and a co-culture system of M0 macrophages with HK-2 cells were established to simulate the renal microenvironment. Experimental results revealed that CHR attenuated the LPS-induced reduction in HK-2 cell viability and suppressed the expression of apoptotic proteins, thereby mitigating cellular injury and apoptosis. Furthermore, CHR effectively inhibited the expression of inflammatory factors TNF-α and IL-6, reducing inflammation in the SA-AKI cellular model. These findings indicate CHR’s potential to ameliorate SA-AKI-induced cellular injury. Additionally, using a murine sepsis model, we validated CHR’s protective effect against SA-AKI *in vivo*, observing significant amelioration of sepsis-induced renal tissue damage consistent with *in vitro* results. Collectively, these data establish CHR as a promising therapeutic agent for SA-AKI.

Network pharmacology analysis identified 78 potential targets of CHR for SA-AKI treatment, with core targets including AKT1, ESR1, HSP90AA1, EGFR, and MAPK3. AKT1, a core serine/threonine kinase within the PI3K/AKT pathway, is inhibited by CHR via the PI3K/AKT/mTOR axis, as evidenced by reduced AKT expression and ameliorated neuropathology in a propionic acid-induced rat autism model (Sharma et al., 2022). This neuroprotective effect confirms AKT1 as a key CHR target. Notably, aberrant AKT activation promotes IκBα kinase activity, leading to IκBα degradation and subsequent NF-κB pathway activation–a pro-inflammatory mechanism. ESR1 (estrogen receptor alpha) modulates inflammatory cytokine production and suppresses inflammation, including via regulation of the TLR4/NF-κB/STAT1 pathway in SA-AKI mice (Zhang et al., 2022). HSP90AA1 functions as a core danger-associated molecular pattern in sepsis (Fitrolaki et al., 2016). EGFR, the receptor for epidermal growth factor and TGF-α, represents a potential anti-inflammatory target; blockade of pro-inflammatory cytokine binding to EGFR may contribute to therapeutic effects (Liu et al., 2021). MAPK3 activation drives NF-κB nuclear translocation, amplifying inflammatory factor release and accelerating SA-AKI progression (Luo et al., 2021). Collectively, these core targets are critically involved in SA-AKI pathogenesis, predominantly through NF-κB signaling pathway regulation.

KEGG pathway enrichment analysis revealed that key signaling pathways implicated in CHR’s therapeutic effect against SA-AKI include the IL-17, cGMP-PKG, calcium, and NF-κB signaling pathways. Notably, the NF-κB pathway is critically involved in both inflammatory responses and macrophage regulation. NF-κB, a transcription factor with broad regulatory functions, typically resides in the cytoplasm as an inactive trimeric complex comprising p65, p50, and IκBα (Chen et al., 2022). Upon cellular stimulation, IκBα undergoes phosphorylation and ubiquitination, leading to its proteasomal degradation (Prescott et al., 2021). This releases the core transcription factor p65, which is subsequently phosphorylated and translocates into the nucleus. Nuclear p65 then initiates and regulates the transcription of genes central to immune and inflammatory responses (Feng et al., 2022).

In this study, follow-up experiments were conducted to determine whether CHR confers protection against SA-AKI by modulating macrophage polarization and to elucidate the underlying mechanism. Experimental results demonstrated that CHR treatment significantly decreased the release of inflammatory factors TNF-α and IL-6 and reduced the expression of M1 macrophage-associated markers (CD86 and iNOS). Conversely, it increased the expression of the anti-inflammatory factor TGF-β and M2 macrophage markers (CD206 and Arg-1). These findings indicate that LPS-treated macrophages predominantly exhibited an M1-polarized phenotype. CHR intervention, however, effectively suppressed M1 polarization while promoting polarization toward the M2 phenotype.

Building upon network pharmacology predictions, this study validated key NF-κB pathway components. LPS stimulation activated the NF-κB signaling pathway, inducing phosphorylation and nuclear translocation of the p65 subunit. CHR treatment, however, suppressed LPS-induced p65 phosphorylation and nuclear translocation, thereby inhibiting NF-κB pathway activation. This inhibitory effect was corroborated by IF analysis. As NF-κB activation drives inflammatory mediator release that promotes M1 macrophage polarization and inflammatory cascades, CHR-mediated NF-κB suppression consequently reduced M1 polarization while favoring M2 polarization. This shift ultimately attenuates pro-inflammatory cytokine release, promotes inflammation resolution, and ameliorates SA-AKI.

Collectively, these findings demonstrate that CHR confers protection against SA-AKI by modulating macrophage polarization within the inflammatory microenvironment. Mechanistically, CHR suppresses NF-κB pathway activation, thereby promoting M2 macrophage polarization while inhibiting M1 polarization. This phenotypic shift reduces inflammatory factor expression, ultimately attenuating renal injury in SA-AKI.

Despite these findings, our study has limitations. First, the complex pathogenesis of SA-AKI involves multiple cell types and organ systems, while our focus remained primarily on macrophages and renal tubular cells. Second, although we identified CHR as protective against SA-AKI at a selected dose, a comprehensive evaluation of its dose-response relationship and potential toxicity is essential for therapeutic validation and represents a critical next step following this mechanistic exploration. Future studies will address these aspects to better inform potential clinical translation.

## 5 Conclusion

In conclusion, this study demonstrates that CHR attenuates SA-AKI by suppressing NF-κB pathway activation in macrophages, thereby promoting M2 polarization while inhibiting M1 polarization, ultimately ameliorating renal damage.

## Data Availability

The original contributions presented in the study are included in the article/supplementary material, further inquiries can be directed to the corresponding authors.

## Abbreviations

CHR, Chrysophanol; SA-AKI, Sepsis-associated acute kidney injury; ELISA, Enzyme-linked immunosorbent assay; IL, Interleukin; TNF, Tumor necrosis factor; TGF, Transforming growth factor; HE, Hematoxylin-eosin; LPS, Lipopolysaccharide; HK-2, Human kidney-2; THP-1, Tohoku hospital pediatrics-1; NF-κB, Nuclear factor-kappa B; iNOS, Inducible nitric oxide synthase; Bcl2, B-cell lymphoma-2; BAX, BCL2-associated X protein; CRRT, Continuous renal replacement therapy; SDS-PAGE, Polyacrylamide gel electrophoresis; PVDF, Polyvinylidene fluoride; HRP, Horseradish Peroxidase; PMA, Phorbol 12-myristate 13-acetate; Arg1, Arginase-1.
